# Will New Variants Emerge after Delta and Omicron?

**DOI:** 10.14336/AD.2022.0307

**Published:** 2022-10-01

**Authors:** Zhen Yang, Shuo Zhang, Yu-Ping Tang, Shi-Jun Yue, Ding-Qiao Xu, Rui-Jia Fu, Sai Zhang, Qi-Ling Liu

**Affiliations:** ^1^Key Laboratory of Shaanxi Administration of Traditional Chinese Medicine for TCM Compatibility, and State Key Laboratory of Research & Development of Characteristic Qin Medicine Resources (Cultivation), and Shaanxi Key Laboratory of Chinese Medicine Fundamentals and New Drugs Research, and Shaanxi Collaborative Innovation Center of Chinese Medicinal Resources Industrialization, Shaanxi University of Chinese Medicine, Xi’an, Shaanxi, China.; ^2^School of Public Health, Shaanxi University of Chinese Medicine, Xi’an, Shaanxi, China.; ^3^School of Clinical Medicine (Guang’anmen Hospital), Beijing University of Chinese Medicine, Beijing, China.

**To the editor,**

The coronavirus disease 2019 (COVID-19) emerged by the 2019 year’s end and became pandemic around the world; as of 5 March 2022, Cumulative confirmed cases globally are 443,777,811 with 5,989,860 deaths (https://coronavirus.jhu.edu/map.html). Contamination with this infection makes individuals foster different respiratory indications, going from gentle manifestations to extreme hypoxia in intense respiratory misery condition. Due to the expanding pace of case notification in China and internationally, a worldwide wellbeing crisis was declared by the WHO Emergencies Committee on January 30, 2020 [1].

Coronaviruses are positive single-stranded RNA viruses with an envelope and are approximately 30 kb in size [2]. They are essentially partitioned into four genera: α, β, γ and δ of gene structure [3]. Coronavirus consists of four primary proteins: spike (S), membrane (M), envelope (E) and nucleocapsid (N) [4]. One of the spike proteins, which decides the diversity and host orientation of the coronavirus, consists of two functional modules; the S1 subunit binds to host cell receptor and the S2 subunit is responsible for the fusion of the virus and the cell membrane [5]. In addition, S1 contains a nitrogen-terminal structural domain (NTD) and a receptor-binding structural domain (RBD), which interacts with the cellular receptor angiotensin-converting enzyme 2 (ACE2), allowing the virus to invade the body and cause respiratory infections [6].

The large-scale epidemic and high infectivity of the virus are difficult to control quickly. The nature of high infectivity may be due to its strong replication fitness leading to higher viral loads and more variants. This results in massive viral replication, expanding the opportunity of adaptive mutations occurring. The continued development of new coronavirus variants has drawn close attention to virus adaptation, transmission, and disease modification. As of 18 December 2021, WHO has classified the new coronavirus variants into five “variants of concern” (VOC) (Table 1) and two ‘variants of interest’ (VOI). The current “variants of concern” are Alpha (B.1.1.7), Beta (B.1.351), Gamma (P.1), Delta (B.1.617.2) and Omicron (B.1.1.529), which were first discovered in UK, South Africa, Brazil, and India, where the earliest recorded samples of Omicron were also found in South Africa. Emerging variants not only have profound impacts on clinical, symptomatic, therapeutic, and public health strategies, but also have a series of negative impacts on socioeconomics and people's lives. How long will the pandemic continue and where is COVID-19 headed? Will new variants continue to emerge after the latest appearance of Omicron?

**Table 1:** List of the most representative VOCs. Summary of the reported SARS-CoV-2 variants divided by clade, first detection and Spike mutations.

	*Alpha*	*Beta*	*Gamma*	*Delta*	*Omicron*
**Scientific name**	**Pango:** B.1.1.7 **Nextstrain:** 20I/B 501Y.V1 **GISAID:** GRY, GR/501Y.V1	**Pango:** B.1.351 **Nextstrain:** 20H/501Y.V2 **GISAID:** GH/501Y.V2	**Pango:**P.1 **Nextstrain:** 20J/501Y.V3 **GISAID:** GR/501Y.V3	**Pango:** B.1.617.2 **Nextstrain:** 21A/S:478K **GISAID:** G/452R.V3	**Pango:** B.1.1.529 **Nextstrain:** 21K **GISAID:** GR/484A
**First detection**	September 2020 UK	May 2020 South Africa	November 2020 Brazil	October 2020 India	November 2021 South Africa
**Spike mutations**	Del69/70, Del144, N501Y, A570D, D614G, T716I, P681H, S982A, D1118H	Del 241/243, 242-244 del, L18F, D80A, D215G, R246I, K417N, E484K, N501Y, D614G, and A701V	L18F, T20N, P26S, D138Y, R190S, K417T, E484K, N501Y, D614G, H655Y, T1027I, V1176F	Del 157/158, T19R, L452R, T478K, D614G, P681R, D950N	Del143/145, Del69/70Del211, ins214EPE, A67V, T951, G142D, L212I, G339D, S371L, S373P, S375F, K417N, N440K, G446S, S477N, T478K, E484A, Q493R, G496S, Q498R, N501Y, Y505H, T547K, D614G, H655Y, N679K, P681H, N764K, D796Y, N856K, Q954H, N969K, L981F

## SARS-CoV-2 variant Delta

**Figure 1.**
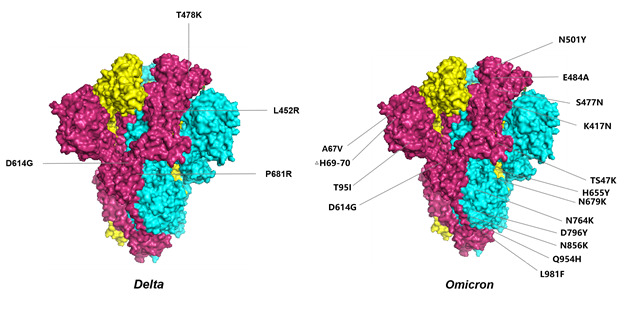
Significant mutations located at the S protein in Delta and Omicron.

In late 2020, variant B.1.617.2 was first distinguished in Maharashtra and spread across India, overtaking the original spectrum including B.1.617.1 (Kappa) and B.1.1.7 [7]. Two critical amino acid substitutions (L452R and E484Q) have been reported in the early viral sequences found in India in the spike in glycoprotein receptor-binding domain, the major immunodominant area and the district engaged with ACE2 binding [8]. Substitution of L452R has been displayed to diminish the binding of several monoclonal antibodies and recovery plasma, and L452R has appeared independently in several pedigrees recommending a role in immune evasion and/or viral adaptation. Also, one of the characteristic mutations of the B.1.427/B.1.429 lineage is L452R. Several studies have shown that the L452R mutation may be associated with enhanced interaction of the human angiotensin-converting enzyme 2 (ACE2) receptor for the COVID-19 stinger protein, allowing the stinger protein(s) to attach to the ACE2 receptor with higher affinity and most likely leading to increased infection rates [9]. L452R was also identified in the California variant, which was related with an increased viral load and an increased transmission rate of approximately 20% [10]. In addition, it was related with increased ACE2 binding, expanded infectivity, and a 3-6-fold decrease in neutralization susceptibility to vaccine-initiated serum in pseudotyped virus (PV) particle experiments [11, 12]. The E484Q mutation was first identified in B.1.351 and P.1 and was subsequently replaced by the functionally similar E484K, which has also appeared independently multiple times in other populations [13]. One more striking mutation of the B.1.617.2 variant is the replacement of P681R, a mutation that contributes to the cleavage of the precursor spinosin into activated spinosin S1 and S2, which would permit better fusion and integration of the virus with the host cell, thus increasing the higher pathogenicity of this variant [14]. This variant of the spike protein contains nine mutations, including five NTD mutations (T19R, G142D, δ156, δ157, R158G), two RBD mutations (L452R, T478K), one mutation near the Flynn cleavage site (P681R) and one mutation in the S2 region (D950N) (Fig. 1) [15]. Due to its rapid spread and potential immune evasion, it was classified as a VOC by the World Health Organization on May 11, 2021 (www.who.int/publications/m/item/weeklyepidemiological-update-on-covid-19).

A few normal indications of the Delta variant are pyrexia, tussis, anhelation, vomiting, diarrhea, sore throat and cephalalgia, while other symptoms include myalgia, ageusia, anosphrasia, fatigue and nasal leakage [16]. Studies have shown that the Delta variant and the Alpha variant have similar symptoms, but Delta patients who mutate develop the virus faster and have higher viral load in their airways. the Delta variant uniquely causes hearing loss and gangrene and more severe blood clots, but rarely causes cough and loss of smell (https://asm.org/Articles/2021/July/How-Dangerous-is-the-Delta-Variant-B-1-617-2). The observation that the B.1.617.2 S protein can cause more intercellular fusions than WT S may indicate that the B.1.617.2 protein is able to cause more tissue damage than the previous variant and is therefore more pathogenic, or that virus transmission through syncytium formation contributes to the efficient transmission of this variant between and within hosts [17]. Bernal et al. observed that a single-dose of BNT162b2 was 30.7% successful against Delta; two doses of BNT162b2 were viewed as 88% viable against Delta; ChAdOx1 nCoV-19 was found to be 67% effective against delta; and the two-dose viability of the ChAdOX1 vaccine was decreased to 59.8% [18].

## SARS-CoV-2 variant Omicron

On 26 November, 2021, WHO identified variant B.1.1.529 as a variant of concern, naming it Omicron, a decision made by the WHO Technical Advisory Group on Virus Evolution (TAG-VE) after observing that Omicron has several mutations that could affect its rate of transmission or the severity of the infection it causes (www.who.int/en/activities/tracking-SARS-CoV-2-variants/). This mutation was identified in samples gathered in Botswana on 11 November 2021, and in South Africa on 14 November 2021, and its occurrence was associated with a sensational expansion in COVID-19 infection in South Africa. The variation in the spike protein is determined by 30 mutations, 15 of which happen in the receptor binding domain, as well as three small deletions and one small insertion [19]. Sequencing of VOC Omicron uncovered roughly 50 changes in its genome, including in excess of 30 mutations on the stinger protein used to tie to the host cell [20]. This has led, to some extent, to decreased vaccine efficacy, immune escape and increased transmissibility. A total of 15 mutations were distinguished in RBD, of which 4 mutations, K417N, Q493R, N501Y and Y505H, affecting 9 key residues, may have extraordinarily enhanced the binding ability of the virus to hACE2 and increased the infectivity of the virus [21]. The N501Y mutation was recently identified in the Beta and Gamma variants and is a far-reaching mutation. N501Y mutant S proteins bind to the hACE2 receptor with 9-fold higher affinity than wild-type S proteins [22]. The open conformation of the N501Y spine protein is related with more effective viral section and disease [23]. Mutation K417N is also an important mutation found in the RBD region, and it is also detected in the B.1.351 variant. The increased affinity of this variant for ACE2 prompts expanded infectivity and pathogenicity of SARS-CoV-2 [24]; Q493R is the only mutation so far causing resistance to bamlanivimab and etesivimab, with a >100-fold reduction in sensitivity to the combination of the two drugs in pseudoviral neutralization assays; in addition, the Q493 mutation increases the binding affinity for angiotensin-converting enzyme 2 [25].

As the most highly mutated SARS-CoV-2 variant, the Omicron variant may spread more rapidly, be more infectious, and escape immunization more easily than previous variants [26]. Omicron has been accounted for to cause asymptomatic infections or less symptomatic ailments, with common early symptoms including sore throat, headache, runny nose, body aches, fever, and exhaustion/weakness [27]. Notably, the majority of reported individuals infected with the Omicron variant have been vaccinated. In addition, the transmission rate of Omicron virus is 5 times higher than that of delta virus [28]. Omicron virus might be two times as liable to escape from the current COVID-19 vaccine compared to Delta virus [29]. Laboratory data suggest that in vaccinated individuals, Omicron has a significantly lower neutralizing antibody response compared to the initial COVID-19 virus or Delta virus variants, although booster doses improve neutralizing activity [30, 31]. Nick et al. showed that the Omicron variant vaccine for symptomatic disease was significantly less effective than the Delta variant vaccine. Seronegative activity was reduced 20 to 40-fold in those receiving two doses of BNT162b2 vaccine contrasted with early pandemic viruses and no less than 10-fold compared to Delta viruses [32]. The Omicron variant is growing rapidly in many countries, Gardner et al. have shown that neutralizing antibody titers decrease approximately 40-fold, dramatically eroding the protective effect of the vaccine and increasing the relative risk of infection and symptomatic disease by more than 4-fold and the risk of hospitalization by 2-fold. Thus, this variant is probably going to spread more rapidly than the delta variant, particularly in highly immunized populations [33]. On the other hand, there is evidence that Omicron has a lot higher pace of asymptomatic infections, possibly as high as 80-90% [34]. However, vaccination remains the more effective method for targeting variant viruses at this time, and sera from immunized people neutralize the B.1.1.529 variant to a lot of lesser degree than the other variants analyzed. The ability to neutralize B.1.1.529 stayed best in sera from super-immunized individuals (tainted and vaccinated or vaccinated and infected) [35].

## After Delta and Omicron, will new variants emerge?

A common public concern after the emergence of each new variant is whether SARS-CoV-2 has arrived at its evolutionary limit, and will SARS-CoV-2 proceed to mutate and produce new variants after the more pathogenic and transmissible Delta and Omicron variants? Why does the new coronavirus mutate? Mutations, in fact, not only viruses, but any microorganism, any living organism, will undergo some changes in the process of its occurrence, development and evolution. The genes of any pathogen, including viruses, are not unchanging; they will change in different ways throughout history, and mutations will occur. Compared to DNA formed by two strands paired with each other, the single-stranded RNA genome of a new coronavirus has a higher probability of producing mutations or variations. However, these constant and massive mutations of viruses are not random, but are affected by changes in the environment and natural conditions. The mutation of the virus will generally be carried out in the direction of “weaker virulence and stronger transmissibility”. This is because from the perspective of survival evolution strategy, the goal of virus evolution is not to kill the host, but to "survive" and expand its "territory", which can only be achieved through the spread of more hosts. If the virulence increases after the mutation, the transmissibility tends to weaken, and the virus is eliminated as the host dies.

Although Omicron virus infections have risen sharply, high levels of Delta infections still exist in numerous countries. It is unclear how Delta and Omicron will interact; one possibility is that Omicron will restrain the spread of Delta by triggering a neutralizing immune response to Delta in populations infected with Omicron. In individuals infected with Omicron, there was an increased ability to neutralize the Delta strain, making them less likely to be reinfected with Delta. This will rely upon whether Omicron virus is indeed less pathogenic than Delta virus. If that is the case, the rate of COVID-19 severe disease will be decreased, and the infection might shift to be less devastating to individuals and society [36].

Viruses generally evolve in much the same way Darwin did, including hereditary variety, natural selection, and survival of favorable types [37]. Examples such as myxomatosis in Australia support the idea that viruses evolve with a trade-off between virulence and transmissibility, with many viruses evolving toward a middle route that is more conducive to transmission. For example, a long-term transmissible virus (human immunodeficiency virus [HIV]) has some selective advantage, provided its effective transmission rate is relatively low [38]. As the virus spreads from one host to another, it is vital to lessen the tendency of the virus to kill the parasitifer or vector. In the case of the COVID-19, the virus may continue to evolve to better adapt to human populations, and the emergence of new variants may repair these mutations, thereby increasing infectivity and resistance to host immunity. On the other hand, for a new virus to coexist better with its host, it will select for mutations that attenuate the pathogenicity of the current SARS-CoV-2 (relatively reduced transmissibility and pathogenicity). Several mutations have been suggested to exist, such as P323L, L37F, G251V, and Q27stop, which are hypothesized to reduce disease severity. Therefore, like other zoonotic viruses, SARS-COV-2 is expected to gradually adapt and turn out to be pathogenic in humans [39].

The COVID-19 pandemic may become normalized, especially after the emergence of Omicron. Although Omicron mutated viruses have spread globally, early evidence suggests that Omicron is less harmful than Delta. Lewnard et al. showed that contrasted with Delta, patients infected with the Omicron variant were 53% less likely to be hospitalized with symptoms, 74% less likely to be admitted to ICU and 91% less likely to die from the disease; patients had a 70% shorter length of stay (median length of stay for Omicron was 1.5 days; Delta was 5 days); and 90% of patients were discharged within three days. The patients were able to leave the hospital in less than three days [40]. This is basically consistent with the findings of scholars in Britain, South Africa and other places. The COVID-19 pandemic will eventually end, but it will take time. It may also persist, but its spread will be predictable. Given the history of other viral respiratory infections (e.g., the 1918 influenza pandemic) that eventually ended, people were treated with common medications and flu vaccines, and those viruses were not fatal to humans. The COVID-19 epidemic continues to develop, and the virus is in a constant state of mutation, and it is likely that other new variants will keep on arising after Omicron. However, in order to survive for a long time, viruses also need to evolve and find a better symbiosis with their hosts by reducing their pathogenicity and infectivity to get infinitely closer to them.

At present, improving immunity through vaccination is still an effective way to control the pandemic. The U.S. vaccination rate for COVID-19 is nearly 70%, and the three-dose vaccination rate is 22%; the U.K. has recently seen more than 200,000 new confirmed diagnoses per day, but no significant increase in disease deaths. The reason for this is that the UK has an 80% vaccination rate for COVID-19, a three-dose vaccination rate of over 50%, and relatively adequate medical resources. In Asia, Singapore has a high vaccination rate of over 90% for two or more doses of the COVID-19 vaccine, and the disease mortality rate is currently controlled at a very low level; Israel has recently added more than 10,000 new diagnoses every day, but the fatality rate is very low. The main reason is that the vaccination rate is high, with a 50% rate of three doses. In addition, the Israeli government stipulates that people over 60 years old must be vaccinated with the third shot vaccine. Vaccines play significant role in COVID-19 outbreak control. Although the vaccine barrier has not completely prevented the spread of the virus, it has made the virus significantly less virulent and reduced the rate of illness and death among those infected. At the same time, crowd control, maintaining social distance, wearing masks, hand washing, rapid nucleic acid testing, quarantine, isolation and other epidemic prevention measures are also key prevention aspects to control the outbreak.
